# Supplementary material for: Rapid regulation of vesicle priming explains synaptic facilitation despite heterogeneous vesicle:Ca2+ channel distances
Source: eLife. 2020 Feb 20;9:e51032. doi: 10.7554/eLife.51032 (PMC7145420; doi:10.7554/eLife.51032)
Supplement: Figure 1—source data 2. [file elife-51032-fig1-data2.zip › Code Manual Centering AZs.docx]

**How to use the code for centering single AZ STED images of Unc13A**

The procedures described here were performed using MATLAB R2016b on a Windows 7 PC. For further details, please also see comments in the code files.

For our purposes, we used two multipage 8-bit grayscale .tif-Files with an x,y-resolution of 51x51 pixels containing 524 or 586 single images, respectively. Place the script files ‘*Center_pkf.m*’ and ‘*peakfinder.m*’ into the same working directory and set it as the “Current Folder” using the internal MATLAB file navigator.

Then, run the script *Center_pkf* with the following command:

*Center_pkf(filename, writeIO);*

Here, the input *‘filename’* needs to be the path and filename of your .tif stack file, e.g. (including apostrophes):

*‘C:\MATLAB\01_Unc13A.tif’*

The input ‘*writeIO’* needs to be *1* if you want the output to be written into the current directory, or *0* otherwise. In case you set it to *1*, two files will be written to the current directory: ‘*centered.tif*’, containing all AZs with a detected peak, centered to the image space, as well as ‘*avg_centered.tif*’, containing the average image of ‘*centered.tif*’ scaled between 0 and 255 gray values.

If your results are undesirable, especially if the code returns many error messages like “NO PEAKS DETECTED IN IMAGE” followed by the number of the current slice, you can try altering the following parameters in *Center_pkf.m*, particularly the value of *‘peakfinder_thresh1’*:

*peakfinder_edge = 1; % in px; This excludes an edge of the evaluated image*

*peakfinder_dist = 5; % in px; This ensures a minimum distance between clusters*

*peakfinder_thresh1 = 25; % in grey values; Threshold of background subtraction*
