# Supplementary figures and images for: Rapid regulation of vesicle priming explains synaptic facilitation despite heterogeneous vesicle:Ca2+ channel distances
Source: eLife. 2020 Feb 20;9:e51032. doi: 10.7554/eLife.51032 (PMC7145420; doi:10.7554/eLife.51032)

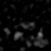

Supplement: Figure 1—source data 2. [file elife-51032-fig1-data2.zip › allAZs_STED_dataset1_Unc13A.tif]

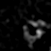

Supplement: Figure 1—source data 2. [file elife-51032-fig1-data2.zip › allAZs_STED_dataset2_Unc13A.tif]

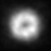

Supplement: Figure 1—source data 2. [file elife-51032-fig1-data2.zip › avgAZ_STED_dataset1_Unc13A.tif]

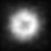

Supplement: Figure 1—source data 2. [file elife-51032-fig1-data2.zip › avgAZ_STED_dataset2_Unc13A.tif]

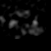

Supplement: Figure 1—source data 2. [file elife-51032-fig1-data2.zip › centeredAZs_STED_dataset1_Unc13A.tif]

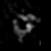

Supplement: Figure 1—source data 2. [file elife-51032-fig1-data2.zip › centeredAZs_STED_dataset2_Unc13A.tif]
