# Supplementary material for: Rapid regulation of vesicle priming explains synaptic facilitation despite heterogeneous vesicle:Ca2+ channel distances
Source: eLife. 2020 Feb 20;9:e51032. doi: 10.7554/eLife.51032 (PMC7145420; doi:10.7554/eLife.51032)

**Readme file for scripts (Kobbersmed at al.)**

**Prior to simulations**

All simulations were carried out in Matlab (R2018b) on a computer grid allowing parallel computing.

- Before simulations the calcium calculator CalC needs to be installed in the same folder as the scripts (the outermost folder). The program as well as a manual on installation and usage can be found here: <https://web.njit.edu/~matveev/calc.html>. After installation, remember to set permissions to allow execution of CalC. Also make sure that the path to the CalC program is correct in the bottom of the RunCalC_det.m script. Otherwise no calcium files will be created and the Matlab script will return an error as it cannot open the calcium file. CalC version 6.8.6 for Mac OS was used in the simulations presented in the paper.
- As the scripts are sorted in folders (Common, Deterministic, Stochastic, etc.) remember to add all folders to the search path. This can be done by calling *add_path* in Matlab in the outermost simulation folder.

**Calling a single simulation**

The script *test_run.m* in the main folder defines all relevant parameters for (both a deterministic and a stochastic) simulation of the unpriming model with the best fitted parameters. As default it runs 200 repetitions of the stochastic simulation. If more is needed use *run_more_reps.m*.

In order to call other setups, the following functions are relevant:

*parameter_choices.m*: Given a model choice and initial parameters, this script

*testing_the_system.m*: Calls an exocytosis run.

The following scripts are the outermost functions for calling the simulations in single simulations, optimisation results, fitting routines, etc.

| **Function** | **Description** | **Input variables** |
| --- | --- | --- |
| *parameter_choices.m* | Given a model choice and initial parameters for that specific model, this script provides an input vector of parameters to be fed into *‘model_parameters_DET’.* Also provides an initial result file name (modified in other scripts).  Output: *par_init, savefilename.* | *par_free*: Model parameters specific for the chosen model.  *model_type:* Choice of model.  *iteration:* Number used to name multiple files when running many repetitions.  *rand_ves_on_off:* In this script only used when ==1000. For good estimation of pVr it set num_bins:=1000 and num_ves :=1e12. |
| *testing_the_system.m* | Calls a single run of calcium and det./stoch. exocytosis simulation (depending on input choice).  Output: Results from simulation e.g. amplitudes, PPRs, pVrs, states from simulation, traces, etc. | *stoch_on_off*: Exocytosis simulation choice. Deterministic (==0), stochastic (==1), or both (==2).  *rand_ves_on_off:* Vesicle placement. See *determ_vesicle_distances.m*.  *CalC_on_off:* 0: No calcium simulation (if calcium files are already generated). 1: Delete old files and simulate new. >1: Simulate calcium without deleting old files.  *par_init*: Parameter vector from *parameter_choice.m*  *CaExtracellular:* Extracel. calcium in simulation.  *save_data:* Defines how many results are saved. See bottom of *simulation_call_stoch.m* and *simulation_call_det.m*. If ==3 only 20 stochastic simulations. If ==66 100 simulations (used when running many reps in sets of 100).  *savefilename:* Part of result file name generated by *parameter_choices.m.*  *save_calc_loc:* 1 or 2. Two choices of folders to save calcium files. If ==2, simulation time is 163.5 ms.  *pVr2_hack:* If ==1, new vesicles are put into the system before AP2 in order to estimate pVr2. |
| *run_more_reps.m* | Calls many repetitions of stochastic simulations in sets of 100. Used for accurate result graphs. Generates temporary result files by iteration, loads these and puts them together in one result file. | *num_iterations:* Number of sets of 100 simulations. Usually ==10.  *par_free*  *model_type:* See *parameter_choices.m*  *CalC_on_off:* See *testing_the_system.m*  *stoch_on_off:* See *testing_the_system.m*  *rand_ves_on_off:* See *testing_the_system.m*  *CaExtracellular:* See *testing_the_system.m*  *save_calc_loc:* See *testing_the_system.m* |
| *fit_model … .m* | Fits the different model with free parameters as noted in script. | Fit files of dual-sensor model (model 2) takes input variables: cooperativity and k_D.  Fit files of unpriming model (model 4) takes input variable: cooperativity.  For optimisations presented in script: *fit_model1_Qmax_numves_krep.m*  *fit_model2_Qmax_kon_s_numves_krep.m*  *fit_model3_Qmax_beta_gamma_delta_numves.m*  *fit_model4_Qmax_kM_unprim_numves_primrate.m* |
| *cost_model … .m* | Used by ‘*fit_model … .m’* scripts. Given a set of parameters, runs deterministic simulation and determines the cost value. | All input parameters are defined in the ‘*fit_model … .m’* scripts.  *pars*: Parameters of the model. (all models)  *save_date:* Given in ‘*fit_model … .m’*. ==0 when running optimisation. ==1 after best parameters are found.  Some models have additional input variables such as cooperativity and k_D. |
| *call_ … .m* | Calling various of the above scripts e.g. single simulations, optimisation results, fitting routines, etc.  These scripts are modified depending on purpose. | None. |

The following figure provides an overview of the major functions called in a single simulation run (scripts for smaller calculations are not depicted):


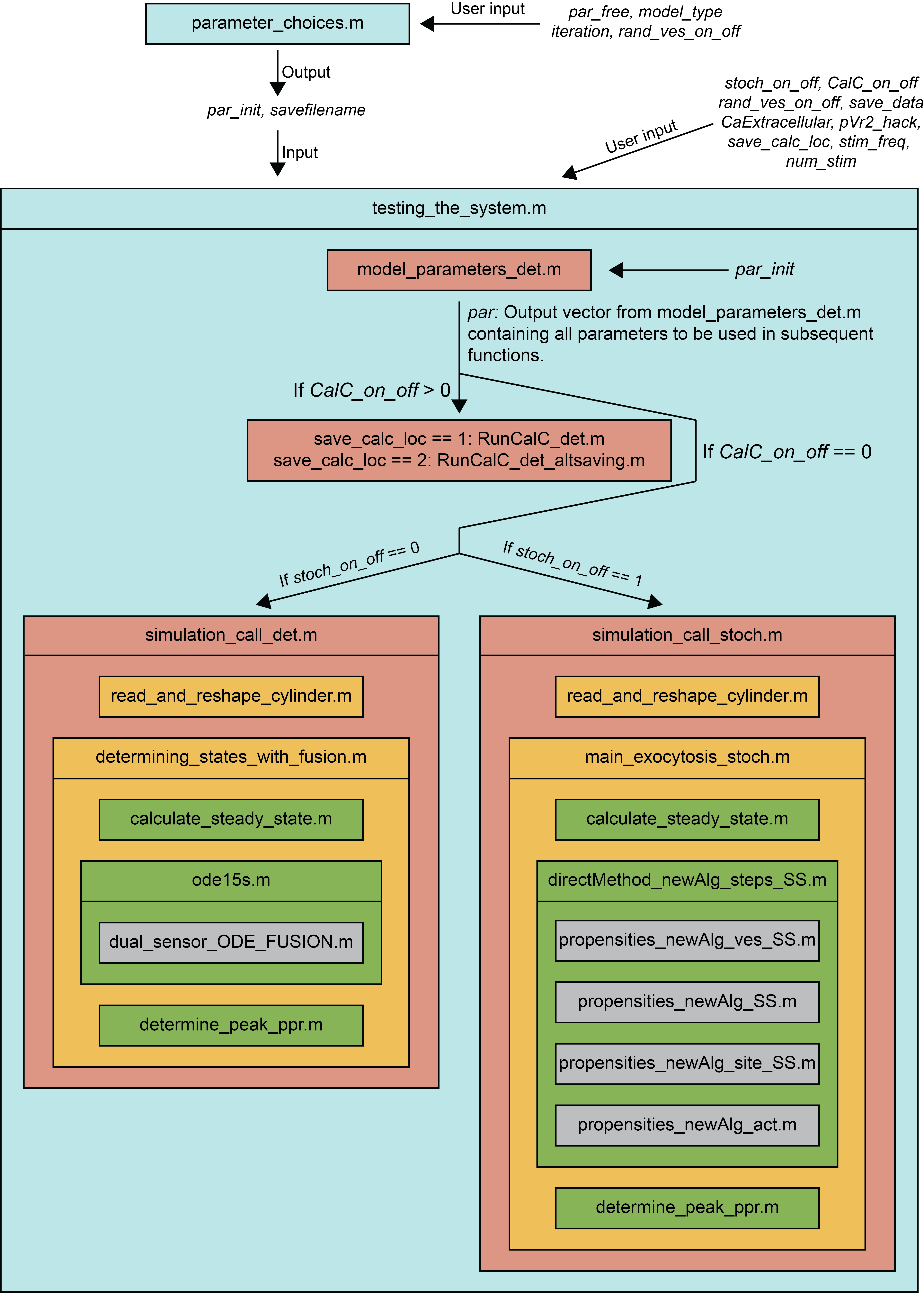

Supplement: Source code 1. [file elife-51032-code1.zip › Matlab_scripts_for_simulation_Kobbersmed_et_al/Readme_simulation_Kobbersmed_et_al.docx]
