# Supplementary material for: Rapid regulation of vesicle priming explains synaptic facilitation despite heterogeneous vesicle:Ca2+ channel distances
Source: eLife. 2020 Feb 20;9:e51032. doi: 10.7554/eLife.51032 (PMC7145420; doi:10.7554/eLife.51032)
Supplement: Transparent reporting form [file elife-51032-transrepform.docx]

***eLife’s* transparent reporting form**

We encourage authors to provide detailed information *within their submission* to facilitate the interpretation and replication of experiments. Authors can upload supporting documentation to indicate the use of appropriate reporting guidelines for health-related research (see [EQUATOR Network](http://www.equator-network.org/%20)), life science research (see the [BioSharing Information Resource](https://biosharing.org/)), or the [ARRIVE guidelines](http://www.plosbiology.org/article/info:doi/10.1371/journal.pbio.1000412) for reporting work involving animal research. Where applicable, authors should refer to any relevant reporting standards documents in this form.

If you have any questions, please consult our Journal Policies and/or contact us: [editorial@elifesciences.org](mailto:editorial@elifesciences.org).

**Sample-size estimation**

- You should state whether an appropriate sample size was computed when the study was being designed
- You should state the statistical method of sample size computation and any required assumptions
- If no explicit power analysis was used, you should describe how you decided what sample (replicate) size (number) to use

Please outline where this information can be found within the submission (e.g., sections or figure legends), or explain why this information doesn’t apply to your submission:

No power analysis was used as this is not the tradition in this field of research. Sample size of the different experiments are found in methods (“Data acquisition and analysis”, “Calculation of mean distance to four nearest neighbors (1-4-NND)”, and “Presynaptic GCaMP recordings & analysis”). Datasets from EM micrographs and the STED images analyzed here (Figure 1) had previously also been used in different analyses in published works as stated in Methods.

**Replicates**

- You should report how often each experiment was performed
- You should include a definition of biological versus technical replication
- The data obtained should be provided and sufficient information should be provided to indicate the number of independent biological and/or technical replicates
- If you encountered any outliers, you should describe how these were handled
- Criteria for exclusion/inclusion of data should be clearly stated
- High-throughput sequence data should be uploaded before submission, with a private link for reviewers provided (these are available from both GEO and ArrayExpress)

Please outline where this information can be found within the submission (e.g., sections or figure legends), or explain why this information doesn’t apply to your submission:

Information on repetitions, outliers, and exclusion/inclusion criteria can be found in figure legends and Methods (“Data acquisition and analysis”, “Calculation of mean distance to four nearest neighbors (1-4-NND)”, and “Presynaptic GCaMP recordings & analysis”).

Sample sizes are defined in the figure legends. Biological replicates correspond to different animals. No technical replications were performed. No outliers were excluded from the given datasets. Data shown in Figure 2 was obtained from 6 individual cells (from 6 animals) and which were stimulated 10 times at each Ca^2+^ concentration. For analysis, the first stimulation per given concentration was excluded (for more details see methods).

**Statistical reporting**

- Statistical analysis methods should be described and justified
- Raw data should be presented in figures whenever informative to do so (typically when N per group is less than 10)
- For each experiment, you should identify the statistical tests used, exact values of N, definitions of center, methods of multiple test correction, and dispersion and precision measures (e.g., mean, median, SD, SEM, confidence intervals; and, for the major substantive results, a measure of effect size (e.g., Pearson's r, Cohen's d)
- Report exact p-values wherever possible alongside the summary statistics and 95% confidence intervals. These should be reported for all key questions and not only when the p-value is less than 0.05.

Please outline where this information can be found within the submission (e.g., sections or figure legends), or explain why this information doesn’t apply to your submission:

Statistical methods of experiments are described in figure legends and Methods (“Electrophysiological data acquisition and analysis”, “Calculation of mean distance to four nearest neighbors (1-4-NND)”, and “Presynaptic GCaMP recordings & analysis”). Statistics of simulations are presented in Results (“Stochastic simulations and fitting of release models”) and in Methods (“Simulation flow”, “Analysis of simulated eEJCs”, “Fitting routine”). Source data with individual observations are provided (listed below).

(For large datasets, or papers with a very large number of statistical tests, you may upload a single table file with tests, Ns, etc., with reference to sections in the manuscript.)

**Group allocation**

- Indicate how samples were allocated into experimental groups (in the case of clinical studies, please specify allocation to treatment method); if randomization was used, please also state if restricted randomization was applied
- Indicate if masking was used during group allocation, data collection and/or data analysis

Please outline where this information can be found within the submission (e.g., sections or figure legends), or explain why this information doesn’t apply to your submission:

No group allocations or randomization was used, as this is not relevant for this kind of study.

**Additional data files (“source data”)**

- We encourage you to upload relevant additional data files, such as numerical data that are represented as a graph in a figure, or as a summary table
- Where provided, these should be in the most useful format, and they can be uploaded as “Source data” files linked to a main figure or table
- Include model definition files including the full list of parameters used
- Include code used for data analysis (e.g., R, MatLab)
- Avoid stating that data files are “available upon request”

Please indicate the figures or tables for which source data files have been provided:

**Figure 1B:** Histogram of docked SV distances to T-bar center – source data file, excel sheet named “Figure 1-Source Data 1.xls”, sheet number one.

**Figure 1E-F:** Average STED image of Unc13A fluorescence (STED dataset 1) and scripts for image analysis– source data zip file, “Figure 1 - Source Data 2.zip”, TIFF image “avgAZ_STED_dataset1_Unc13A.tif”. Fluorescence intensities against distance from the AZ center (F) – source data file, excel sheet named “Figure 1-Source Data 1.xls”, sheet number two.

**Figure 1 – figure supplement 1:** Histogram of docked SV distances to T-bar center – source data file, excel sheet named “Figure 1-Source Data 1.xls”, sheet number three. Average STED image of Unc13A fluorescence and scripts for image analysis– source data zip file, “Figure 1 - Source Data 2.zip”, TIFF image “avgAZ_STED_dataset2_Unc13A.tif”. Fluorescence intensities against distance from the AZ center (F) – source data file, excel sheet named “Figure 1-Source Data 1.xls”, sheet number two.

**Figure 2:** Electrophysiology, measured eEPSC1/2 amplitudes and PPR values (single values, mean and variances) – source data file, excel sheet named “Figure 2-Source Data 1.xls”, sheets number one through three. Scripts for data analysis - source data zip file, “Figure 2 - Source Data 2.zip”, raw data from experiments named “Figure 2 – Source 3.zip.”

**Figure 2 – figure supplement 1:** Best fit parameters of the mEJC used in simulations can be found in “Generating the mEJC for convolution of eEJCs” in Methods, source data file with raw mEJC traces named Figure 2- Source data 4.zip.

**Figure 2 – figure supplement 3:** Raw data from experiments in WT and WT/Ok6-Gal4 recordings at 0.75 and 1.5 mM Calcium named “Figure 2-figure Source data 5.zip”

**Figure 3A:** k-neighbour analysis, determining the average distance to the four nearest neighboring AZs – source data file, excel sheet named “Figure 3-Source Data 1.xls”, sheet number one.

**Figure 3B-C:** Q_max_ value can be found in Table 3 (best fit of single sensor model). All other parameters used in CalC simulations can be found in Table 1.

**Figure 3 – figure supplement 1:** Result summary, best fit curve parameters and data points for each cell in panel D - source data file, excel sheet named “Figure 3-Source Data 1.xls”, sheet number two. Best fit parameters can be found in Table 3.

**Figure 3 – figure supplement 2:** Best fit Q_max_ value for the different models can be found in table 3. All other parameters used in CalC simulations can be found in Table 1.

**Figure 4:** Simulated eEPSC_1_, PPR values (mean and SD) of single sensor model – source data file, excel sheet named “Figure 4-Source Data 1.xls”, sheet number one. Best fit parameters can be found in Table 3.

**Figure 4 – figure supplement 1:** Exploration of PPR values in deterministic and stochastic simulations with and without replenishment – source data file, excel sheet named “Figure 4-Source Data 1.xls”, sheet number two.

**Figure 5:** Example simulations and average simulations of single-sensor model. Simulations were run with best fit parameters, which can be found in Table 3.

**Figure 6:** Simulated eEPSC1, PPR values (mean and SD) of dual fusion-sensor model – source data file, excel sheet named “Figure 6-Source Data 1.xls”, sheet number one. Best fit parameters can be found in Table 3.
**Figure 6 – figure supplement 1:** Simulated eEPSC_1_, PPR values (mean and SD) of dual fusion-sensor model cooperativity 5 – source data file, excel sheet named “Figure 6-Source Data 1.xls”, sheet number two. Best fit parameters can be found in Table 3.

**Figure 7:** Simulated eEPSC_1_, PPR values (mean and SD) of unpriming model – source data file, excel sheet named “Figure 7-Source Data 1.xls”, sheet number one. Best fit parameters can be found in Table 3.

**Figure 7 – figure supplement 1:** Simulated eEPSC_1_, PPR values (mean and SD) of dual fusion-sensor model cooperativity 5 and unpriming model cooperativity 2 – source data file, excel sheet named “Figure 7-Source Data 1.xls”, sheet number two. Best fit parameters can be found in Table 3.

**Figure 7 – figure supplement 2:** Simulated eEPSC_1_, PPR values (mean and SD) of site activation model – source data file, excel sheet named “Figure 7-Source Data 1.xls”, sheet number three. Best fit parameters can be found in Table 3.
**Figure 7 – figure supplement 3:** Simulated PPR values (mean and SD) of unpriming model for different ISIs – source data file, excel sheet named “Figure 7-Source Data 1.xls”, sheet number four. Best fit parameters can be found in Table 3.

**Figure 8:** Example simulations and average simulations of unpriming model. Simulations were run with best fit parameters, which can be found in Table 3.

**Figure 9:** Illustration of model simulations. pV_r_ and Ca^2+^ graphs are from simulations with best fit parameters of the models. Best fit parameters can be found in Table 3.
